# Imaging of Surfaces by Concurrent Surface Plasmon Resonance and Surface Plasmon Resonance-Enhanced Fluorescence

**DOI:** 10.1371/journal.pone.0009833

**Published:** 2010-03-25

**Authors:** Rahber Thariani, Paul Yager

**Affiliations:** Department of Bioengineering, University of Washington, Seattle, Washington, United States of America; Virginia Tech, United States of America

## Abstract

Surface plasmon resonance imaging and surface plasmon induced fluorescent are sensitive tools for surface analysis. However, existing instruments in this area have provided limited capability for concurrent detection, and may be large and expensive. We demonstrate a highly cost-effective system capable of concurrent surface plasmon resonance microscopy (SPRM) and surface plasmon resonance-enhanced fluorescence (SPRF) imaging, allowing for simultaneous monitoring of reflectivity and fluorescence from discrete spatial regions. The instrument allows for high performance imaging and quantitative measurements with surface plasmon resonance, and surface plasmon induced fluorescence, with inexpensive off-the-shelf components.

## Introduction

Laboratories in the developing world may be severely resource-constrained in terms of funding, physical infrastructure, and high-end technical support personnel [1]. Instruments aimed at building the capacity for research in developing countries must therefore be constructed from easily available off-the-shelf components, be simple to maintain, and be highly cost-effective, whilst ideally providing multiple modality capability on the same device. In our laboratory we are investigating instruments and assays for both conducting research and field deployment in resource-poor areas [2], [3].

Surface plasmons are charge density-oscillations created at the interface of two media with dielectric constants of different signs, for example between a noble metal and glass. The resonance of these surface plasmons can occur when the wavevector of p-polarized light incident upon an interface matches the wavevector of the surface plasmons; this matching of wavevectors is characterized by a drop in the photon flux reflected from the interface. Fields associated with surface plasmons extend into the media surrounding the interface and decay exponentially, and are thus sensitive to changes in the media around them [4]. Surface plasmon resonance imaging or microscopy (SPRM), as introduced by Rothenhausler and Knoll in 1988 [5], uses the excitation of surface plasmons to simultaneously interrogate the near-surface refractive index (RI) at multiple sites at a sample surface. The electric field of the surface plasmons can also be used to excite near-surface fluorophores (SPRF) [6]. This technique allows for highly sensitive and localized detection due to surface confinement (the surface plasmon field is evanescent and decays exponentially), enhancement of the incident electric field intensity (up to 80× depending upon the metal layer [7], [8]), and reflection of the excitation light at the interface which reduces unwanted background light. Previous SPRM/SPRF systems using lasers have exhibited good performance utilizing the highly desirable qualities of laser sources such as collimated output, high power and narrow bandwidth [6], [9], [10], [11], [12]. However, to our knowledge imaging in both SPR and SPRF modes simultaneously has not been accomplished, potentially due to speckle artifacts caused by the coherent laser illumination, limiting the ability of these systems to simultaneously track and spatially discriminate between different regions in the sample. In our system the light source used is a conventional laser pointer available at myriad office supply stores. This is coupled with an acoustic transducer to “despeckle” or average out intensity variations due to speckle formation. The use of dark Mylar on the sample flowcell greatly reduces adhesive-derived scattering of the excitation light, allowing for the simultaneous measurement of multiple independent channels on the same flowcell. We demonstrate a highly cost-effective system capable of simultaneously interrogating a sample under SPRM and SPRF, thus spatially discriminating between regions on the same sample. Applications of concurrent SPRM/SPRF include the ability to monitor the the thickness and physiochemical properties of surface layers simultaneously [13], the detection and quantification of multiple analytes onto a surface [14], [15], and to study reactions between multiple proteins interacting at a surface [16]. The concurrent techniques could thus potentially be used to discriminate between specific and non-specific binding events in hetrogenous immunoassays in real-time. In addition, the SPRM and SPRF components can be utilized individually to perform a wide range of biological assays [17], [18], [19], [20], [21].

## Methods

**Figure 1:**
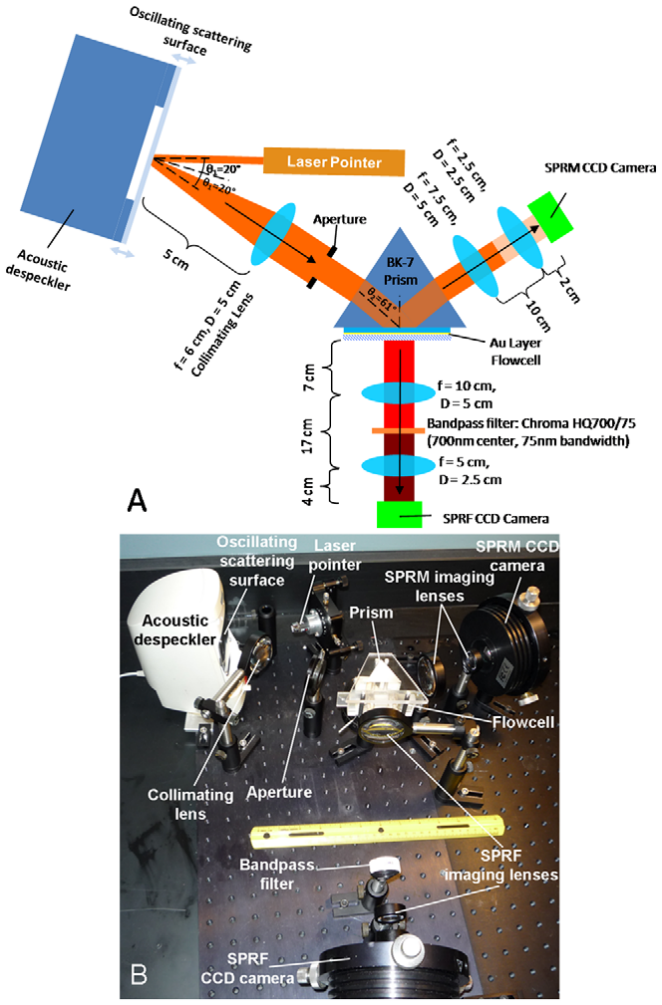
System Overview. Top view schematic (A) (not to scale) and photograph (B) of the instrument with the sample oriented horizontally.

The schematic of our system is shown in Figure 1A with a photograph in Figure 1B, where the Kretschmann configuration [22], [23] is clearly seen. The light source used here is an inexpensive diode pumped solid state laser with the output wavelength centered at 654 nm. The output beam is linearly polarized (50∶1) and is available commercially (Office Depot, Delray Beach, FL). The laser pointer is mounted on an optical stage (Newport Corporation, Irvine, CA) using a simple aluminum mount. The laser can be powered by two 1.5 V silver oxide batteries, but to achieve a more consistent power supply we used a 3 V AC-to-DC adaptor. SPR microscopy instruments driven by coherent illumination can suffer from dramatically reduced image quality due to interference effects, the so-called speckle artifact. We have devised a simple and highly-cost effective method of increasing the quality of the image by an order of magnitude using an acoustic despeckler [24]. In brief, by rapidly changing the optical path length through vibrations of a reflective membrane mounted over an acoustic transducer (ACS43, Altec Lansing, Milford, PA), and by using a suitably long integration time, the speckle artifact is averaged out. Divergent light reflected from the membrane is collimated by a convex lens and directed through an aperture to control beam diameter before entering a BK-7 prism (Melles-Griot, Carlsbad, CA) in the standard Kretschmann configuration, allowing light to couple into the sample flowcell. This sample flowcell typically consists of a Mylar flowcell [25] built upon a glass slide coated with a 45 nm layer of gold. After exiting the prism the light is focused on to a low-cost CCD camera (Orion Starshoot, Watsonville, CA) originally designed for amateur astronomy by means of convex lenses mounted in the Scheimpflug configuration [26]. This forms the SPR microscopy (SPRM) section of the instrument. To enable fluorescence imaging, two convex lenses are placed along an axis normal to the sample flowcell, distally to the incoming light, allowing fluorescence emissions generated from the sample to be focused onto another CCD camera of the same type as above. The placement of the lenses normal to the flowcell allows the focusing of the greatest amount of photon flux of the fluorescence emissions. A bandpass (700/75 nm) filter (Chroma, Rockingham, VT) is placed between the lenses to reduce the impact of any scattered and transmitted laser. This forms the SPRF component of the system. The components for this instrument can be purchased at retail for well under $1000 (USD).

## Results

**Figure 2:**
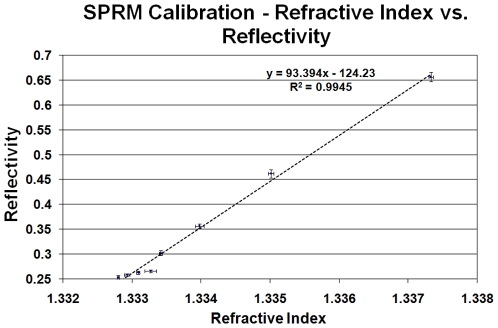
SPRM Calibration. Illustrating the change in reflectivity as a function of refractive index. To compensate for laser power output variations, normalization of the intensity in the region of interest in the flowcell took place against the static adhesive regions surrounding the flowcell. The different refractive indices were obtained by flowing NaCl solutions through the sample flowcell.

**Figure 3:**
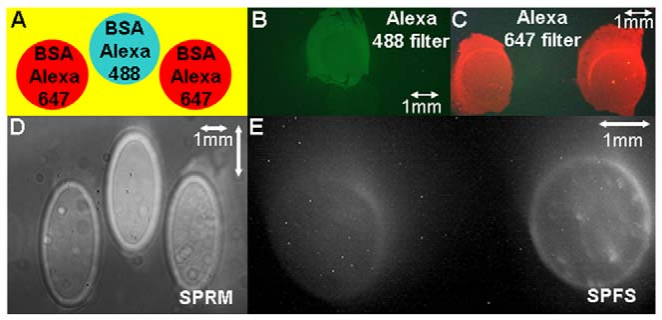
Fluorescence Component Testing. A) Illustrating the schematic of the pattern spotted on the Au-coated slide. B) and C) Images observed through conventional epifluorescence microscopes with filters for Alexa 488 and Alexa 647. D) SPRM Image: all three spots are seen since the SPRM detects the change in the refractive index due to deposition of the BSA. Image is compressed in the x-axis due to the high incident angle of the incoming light. E) SPRF image: only 2 spots, those with Alexa Fluor 647 are seen in the SPRF image.

To calibrate the SPR microscopy system response to different refractive indices (RI) present over the metal surface, we measured the changes in reflectivity with changes in RI utilizing a protocol established previously in our laboratory [22], [23], [24]. Briefly, NaCl solutions of various concentrations are prepared, and the corresponding RI measured in a refractometer. The solutions are then passed through a flowcell attached to the prism and reflectivity measured for each solution. The instrument response for reflectivity vs. RI is shown in Figure 2. We estimate a limit of detection of 1.3×10^−4^ RI units; this is a useful range, in that such a change in RI can be created by less than 5% of a monolayer of bovine serum albumin (BSA) adsorbed to the surface [27]. To test the fluorescence capability of the instrument, a clean glass slide (Fisher Scientific, Pittsburgh, PA) was coated with a 1 nm layer of Cr for adhesion, followed by a 45 nm layer of Au using an electron-beam evaporator (Washington Technology Center, Seattle, WA). The Au surface was cleaned for 10 minutes with a ammonium hydroxide/hydrogen peroxide and DI water solution (1∶1∶5 by volume). Two 1 µL drops of BSA labeled with Alexa Fluor 647, and one 1 µL drop of BSA labeled with Alexa Fluor 488 (Invitrogen, Carlsbad, CA) were deposited upon the gold coated slide forming a test pattern as shown in Figure 3a. Incubation for 45 minutes took place in a sealed Petri dish with a damp paper towel (to avoid solvent loss by evaporation), after which the excess BSA from the spots was pipetted away. A flowcell [25] was then assembled upon the Au-coated slide and rinsed with DI water. The slide was then imaged through conventional epifluorescence microscopy using filters for Alexa Fluor 488 (Figure 3b) and Alexa Fluor 647 (Figure 3c). The flowcell was then placed upon the prism in our instrument and SPRM (Figure 3d) and SPRF (Figure 3e) images taken (integration times of 30ms and 20s respectively). SPR microscopy shows the presence of three spots, whereas the SPRF image only shows the spots labelled with Alexa Fluor 647, since the laser light wavelength (654 nm) does not overlap with the excitation spectra of the Alexa Fluor 488. This demonstrates the label-less detection of the SPR microscope, as well as the specificity of the system in SPR-enhanced fluorescence mode. During our fluorescence measurements, we noticed that regions of the flowcell where the adhesive layer was in direct contact with the gold produced a significant scattering signal even with the presence of the long-pass filter. The adhesive scattering signal was stronger than the signal from a blank channel containing PBS by an order of magnitude, and quite capable of overwhelming the signal from the channels in the flowcell. In order to eliminate this adhesive-based scattering, thus allowing for effective imaging of multiple channels on a single flowcell, customized, adhesive-coated black Mylar was ordered (Fraylock Inc., San Carlos, CA, USA), and was used to construct those areas of the flowcell that were in direct contact with the gold surface.

**Figure 4:**
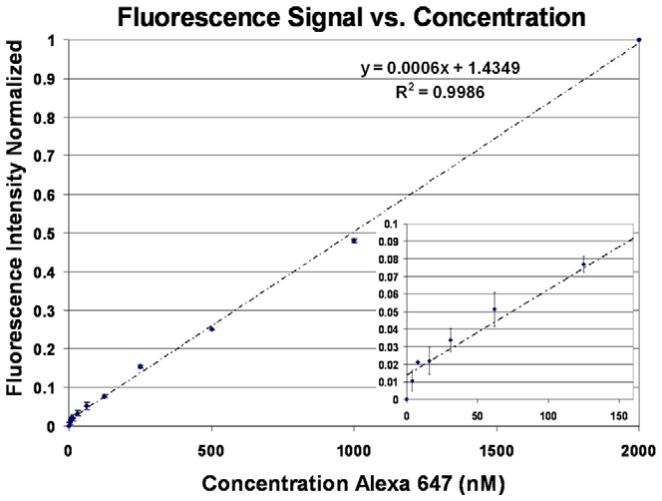
Fluorescence System Calibration. Normalized fluorescence response for different fluorophore concentrations in our instrument. Background subtraction using a control channel on the same flowcell was performed. Inset shows the system response at the lower end of the dilution range.

In order to test the linearity of the fluorescence system with dye concentrations, a Mylar flowcell was assembled over a freshly cleaned Au-coated slide as mentioned above. Different concentrations of Alexa Fluor 647 non-reactive dye were prepared through serial dilutions and passed through the flowcell, from which fluorescence intensity measurements were taken. Apart from the SPRF, the fluorescence signal may have a strong component caused by scattered light. To measure the contribution of any scattered light, the angle of the prism was tuned to be just off the resonance angle, and the drop in fluorescence intensity measured. We observed the scattering to be less sensitive to small changes in angle compared to the SPRF signal (data not shown). Thus difference between the fluorescence intensities at resonance (SPRF + scattering) and just off-resonance (scattering) allows the determination of the fraction of the fluorescence signal due to scattering (∼35%); this was subtracted from our fluorescence measurements. The results from the calibration experiments are illustrated in Figure 4, and we estimate a LOD of 30 nM of Alexa Fluor 647.

## Discussion

In conclusion, an instrument capable of concurrent surface plasmon resonance microscopy and surface plasmon resonance-enhanced fluorescence imaging has been constructed and demonstrated. Whilst sensitive independent instruments are available with these capabilities, our instrument uniquely provides both these capabilities in one compact and highly cost-effective unit. Potential uses include point-of-care diagnostics and lab-on-a-chip applications for global health, where the quantitation of labelled and unlabelled proteins on the same device may be necessary and cost efficacy is highly relevant.
